# Transferrin receptor 1 is required for enucleation of mouse erythroblasts during terminal differentiation

**DOI:** 10.1002/2211-5463.12573

**Published:** 2019-01-08

**Authors:** Mamoru Aoto, Akiho Iwashita, Kanako Mita, Nobutaka Ohkubo, Yoshihide Tsujimoto, Noriaki Mitsuda

**Affiliations:** ^1^ Department of Circulatory Physiology Graduate School of Medicine Ehime University Japan; ^2^ Department of Molecular and Cellular Biology Research Center Osaka International Cancer Institute Japan

**Keywords:** enucleation, erythroblast, hinokitiol, iron, transferrin, transferrin receptor

## Abstract

Enucleation is the process whereby the nucleus is extruded from the erythroblast during late stage mammalian erythropoiesis. However, the specific signaling pathways involved in this process remain unclear. To better understand the mechanisms underlying erythroblast enucleation, we investigated erythroblast enucleation using both the spleens of adult mice with phenylhydrazine‐induced anemia and mouse fetal livers. Our results indicated that both iron‐bound transferrin (holo‐Tf) and the small‐molecule iron transporter hinokitiol with iron ions (hinokitiol plus iron) promote hemoglobin synthesis and the enucleation of mouse spleen‐derived erythroblasts. Although an antitransferrin receptor 1 (TfR1) monoclonal antibody inhibited both enucleation and hemoglobin synthesis promoted by holo‐Tf, it inhibited only enucleation, but not hemoglobin synthesis, promoted by hinokitiol plus iron. Furthermore, siRNA against mouse TfR1 were found to suppress the enucleation of mouse fetal liver‐derived erythroblasts, and the endocytosis inhibitor MitMAB inhibited enucleation, hemoglobin synthesis, and the internalization of TfR1 promoted by both types of stimuli. Collectively, our results suggest that TfR1, iron ions, and endocytosis play important roles in mouse erythroblast enucleation.

**Abbreviations**
CFU‐E: colony‐forming unit‐erythroid
EPO: erythropoietin
holo‐Tf: iron‐loaded transferrin
TfR: transferrin receptor
Tf: transferrin

The process of mammalian erythropoiesis consists of erythropoietin (EPO)‐dependent proliferation of colony‐forming unit‐erythroid (CFU‐E) progenitors and their differentiation into mature erythrocytes. EPO signaling protects the CFU‐E progenitors from apoptosis and stimulates their proliferation and terminal differentiation 1. CFU‐E progenitors subsequently initiate a cascade of morphologically identifiable erythroid precursors, thereby progressing from proerythroblasts to basophilic, polychromatophilic, and orthochromatic erythroblast stages. In the late stages of erythropoiesis, EPO is no longer required 2. Late erythroblasts are characterized by a terminal cell cycle exit, decreased cell size, hemoglobin synthesis, chromatin condensation, cell surface Ter119 expression, and enucleation.

Enucleation is unique to mammalian erythroblasts. During the process, the nucleus, surrounded by the plasma membrane, is extruded from the erythroblast, and many subprocesses are involved, including histone deacetylation 3, 4, actin polymerization 5, 6, cytokinesis 7, cell–matrix interaction 8, cell polarization 9, the downregulation of certain microRNA 10, high long‐noncoding RNA expression 11, and vesicle trafficking 12, 13. Although our knowledge of mammalian erythroblast enucleation is increasing, many aspects of the underlying molecular mechanisms remain unclear.

Transferrin (Tf) is a monomeric serum glycoprotein (~ 80 000 Da) responsible for the delivery of iron ions to most cells, as iron is required for the formation of iron‐containing proteins and the biosynthesis of Fe–S clusters and heme by mitochondria 14. Iron‐loaded Tf (holo‐Tf) uptake is mediated by its binding to a specific cell surface Tf receptor (TfR), followed by holo‐Tf–TfR complex internalization by endocytosis 15. In the late stages of erythroid differentiation, erythroblasts express the TfR protein at high levels. In addition, iron, which is incorporated into the erythroblasts via the Tf–TfR complex, is necessary for hemoglobin synthesis. For an experimental supply of iron into cells, the natural product hinokitiol can be used. Hinokitiol, originally isolated from the essential oil of the *Chamaecyparis taiwanensis* (Taiwan Hinoki) tree, has been shown to bind iron ions and transport them into cells through the plasma membrane 16.

In this study, we used two kinds of erythroblasts for *in vitro* enucleation experiments to determine the mechanisms underlying erythroblast enucleation: (a) erythroblasts from the spleens of adult mice with phenylhydrazine‐induced anemia and (b) erythroblasts from the mouse fetal liver. Erythroblast enucleation was promoted by holo‐Tf and hinokitiol with iron ions (hinokitiol plus iron). Blockage of TfR1 by the anti‐TfR1 monoclonal antibody suppressed the erythroblast enucleation promoted by holo‐Tf or hinokitiol plus iron. These results indicate that TfR1 plays a key role in mouse erythroblast enucleation.

## Materials and methods

### Materials

Human holo‐Tf (208‐18971) and hinokitiol (085‐06251) were purchased from FUJIFILM Wako Pure Chemical Corporation (Osaka, Japan). Recombinant human EPO (873999) was purchased from Chugai Pharmaceutical Co., Ltd. (Tokyo, Japan). Percoll (17‐0891‐02) was obtained from GE Healthcare (Buckinghamshire, UK). FITC‐anti‐mouse CD44 (103006), PE‐conjugated anti‐mouse Ter119 (116208), PE/Cy7‐conjugated anti‐mouse CD71 (113812), Pacific blue‐conjugated anti‐mouse CD45 (103126), biotin‐conjugated anti‐mouse CD45 antibodies (103104), 7‐aminoactinomycin D (7‐AAD; 420404), MojoSort™ Streptavidin Nanobeads (480016), and MojoSort™ Magnet (480019) were purchased from BioLegend (San Diego, CA, USA). The anti‐TfR1 monoclonal antibody R17 208.2 (sc‐65883) was obtained from Santa Cruz Biotechnology (Dallas, TX, USA). Control rat IgM (14‐4341‐85), SYTO16 (S7578), and Cell‐Tak Cell and Tissue Adhesive (354240) were purchased from Thermo Fisher Scientific (Waltham, MA, USA). RITC‐conjugated anti‐rabbit IgG antibody (SA00007‐2) was obtained from Proteintech (Rosemont, IL, USA). MitMAB (ab120466) and anti‐TfR1 antibody (ab84036) were obtained from Abcam (Cambridge, UK).

### Animal experiments and mouse spleen‐derived erythroblast isolation

All animal studies were carried out in accordance with the guidelines of the Ehime University School of Medicine Committee on Animals. All mice were housed in a specific pathogen‐free facility under a 12‐h light/dark cycle with water and standard diet provided *ad libitum*. The purification and *in vitro* culture procedures of mouse spleen erythroblasts were modified based on procedures described previously 17, 18, 19. Briefly, anemia was induced in C57Bl/6 mice at 9–11 weeks of age by infusion with 40 mg·kg^−1^ body weight of phenylhydrazine for two consecutive days 19. Six days later, spleen cells were mechanically dissociated by mashing the mouse spleen. Single‐cell suspensions were prepared by passing the dissociated cells through a 70‐μm cell strainer; suspensions were then layered onto a density gradient of 70% (v/v) Percoll and centrifuged at 2000 ***g*** for 30 min. Cells were labeled with biotin‐conjugated anti‐mouse CD45 antibody, followed by Streptavidin Nanobeads. After washing with 0.5% BSA/PBS, CD45‐negative cells were purified using a magnetic separator as per the manufacturer's instructions. Cells were then characterized by staining with FITC‐anti‐mouse CD44, PE‐anti‐mouse Ter119, PE/Cy7‐anti‐mouse CD71, and Pacific blue‐anti‐mouse CD45 antibodies. 7‐AAD was added for the exclusion of dead cells. Flow cytometry was performed using Gallios (Beckman Coulter, Brea, CA, USA), while data analysis was performed using flowjo software (BD Biosciences, Franklin Lakes, NJ, USA). CD45‐negative cells contained erythroblasts and reticulocytes. For enucleation analysis, CD45‐negative cells (3 × 10^6^ cells·mL^−1^) were incubated at 37 °C in α‐MEM containing 1% BSA with holo‐Tf or 100 μm hinokitiol and/or 33 μm FeCl_3_. Stock solutions of 100 mm hinokitiol and 400 mm FeCl_3_ were made by dissolving in DMSO and 100 mm HCl, respectively. After incubation, the cells were stained with SYTO16 and the PE‐anti‐mouse Ter119 antibody. SYTO16^low^Ter119^high^ cells represented the reticulocyte fraction. The difference in the ratio of reticulocytes before and after culture initiation was calculated as a percentage of enucleation. In assays involving the anti‐TfR1 antibody, the antibody was added to the cells in the medium without holo‐Tf 30 min before culture initiation.

### Biotinylation of transferrin proteins

Human holo‐Tf was biotinylated using a Biotinylation Kit (Sulfo‐Osu; DOJINDO, Kumamoto, Japan), according to the manufacturer's protocol.

### Flow cytometry analysis of transferrin protein binding

The binding of Tf proteins to the cell surface was assessed by flow cytometry. Several K562 cell lines were incubated with 150 μg·mL^−1^ of biotinylated holo‐Tf in PBS (pH 7.4) containing 0.5% BSA for 30 min on ice, followed by washing with 0.5% BSA/PBS and incubation with streptavidin–FITC. The detection of FITC was performed using flow cytometry.

### Hemoglobin assay

Cells (3 × 10^6^) were lysed in 100 μL of Drabkin's reagent, and the hemoglobin content was quantified by spectrophotometric measurement of absorbance at 540 nm using a microplate reader (Bio‐Rad Laboratories, Inc., Hercules, CA, USA).

### Culture and transfection of K562 cells

Human erythroleukemia K562 cells were maintained in RPMI1640 supplemented with 10% FBS. K562‐pcDNA3.1, mouse TfR1, and mouse TfR2 cells were maintained in RPMI1640 supplemented with 10% FBS and 200 μg·mL^−1^ of G418. K562‐pcDNA3.1, mouse TfR1, and mouse TfR2 cells were established by the transfection of K562 cells with pcDNA3.1, pcDNA3.1‐FLAG‐mouse TfR1, and pcDNA3.1‐FLAG‐mouse TfR2, respectively. Transfection of K562 cells was performed using a Cell Line Nucleofector Kit V (Lonza, Basel, Switzerland), according to the general protocol for suspension cell lines described in the manufacturer's instructions. Briefly, cells (1 × 10^6^) were transfected with 5 μg of plasmid DNA as follows. Cells were then transferred to an Amaxa cuvette and nuclear transfection was performed using the T‐16 program. Immediately after transfection, cells were transferred into culture dishes using the recommended plastic pipettes. Forty‐eight hours after transfection, cells were selected using 500 μg·mL^−1^ of G418 and were screened for the detection of stable mouse TfR1 or TfR2 expression using western blot analysis.

### Analysis of the specificity of the anti‐mouse TfR1 antibody R17 208.2

K562, K562‐pcDNA3.1, K562‐mouse TfR1, or K562‐mouse TfR2 were incubated with 1 μg·mL^−1^ anti‐mouse TfR1 antibody (RI7 208.2) or control rat IgM in PBS containing 0.5% BSA for 30 min on ice. This was followed by washing with 0.5% BSA/PBS and incubation with the PE‐anti‐rat IgM antibody. After washing with 0.5% BSA/PBS, cells were analyzed by flow cytometry.

### Purification and culture of mouse fetal liver‐derived erythroblasts

Purification and *in vitro* culture procedures of mouse fetal liver‐derived erythroblasts were conducted using modified versions of previously described procedures 20. Fetal liver cells were isolated from E14.5 C57Bl/6 embryos and mechanically dissociated by mashing in RPMI1640. Single‐cell suspensions were prepared by passing the dissociated cells through 70‐μm cell strainers. The fetal liver cells obtained were then labeled with the biotin‐conjugated anti‐mouse Ter119 antibody, followed by incubation with Streptavidin Nanobeads. After washing with 0.5% BSA/PBS, Ter119‐negative cells were purified with a magnetic separator as per the manufacturer's instructions. Purified cells were then seeded at a density of 1 × 10^6^ cells·mL^−1^. On the 1st day, the purified cells were cultured in Iscove's modified Dulbecco's medium (IMDM) containing 20% FBS, 2 mm l‐glutamine, 10^−4^ m monothioglycerol, and 10 U·mL recombinant human EPO. After 30 h, this medium was replaced with IMDM containing 20% FBS, 2 mm l‐glutamine, and 10^−4^ m monothioglycerol. The erythroblasts were then seeded at a density of 2.5 × 10^6^ cells·mL^−1^. Erythroblasts were characterized by staining with PE‐anti‐mouse Ter119 antibody, PE/Cy7‐anti‐mouse CD71 antibody, and SYTO16, while 7‐AAD was added for the exclusion of dead cells.

### Gene silencing

Mouse TfR1 siRNA #1 (SASI_Mm01_00122815), mouse TfR1 siRNA #2 (SASI_Mm01_00122818), and negative control siRNA (MISSION siRNA Universal Negative Control #1) were purchased from Merck KGaA (Darmstadt, Germany). The sequences of the GFP siRNA were 5′‐GGCUACGUCCAGGAGCGCACC‐3′. Transfection of mouse fetal liver‐derived erythroblasts was performed with a ‘Cell Line Nucleofector kit V’ according to the general protocol for suspension cell lines described in the manufacturer's instructions. Briefly, 1 × 10^6^ cells were transfected with 5 μg of siRNA as follows. The cell suspension was transferred to an Amaxa cuvette, and nuclear transfection was performed using the X‐001 program. Immediately after transfection, the cells were transferred with the recommended plastic pipettes into the culture dishes. Thirty hours after transfection with siRNA, cells were used for the experiments. The efficiency of the transfection was ~ 56% as assessed separately from the transfection of siRNA and by the transfection of pmaxGFP supplied from the kit.

### Immunofluorescence analysis

Mouse spleen‐derived erythroblasts were adhered at 4 °C for 30 min in the presence or absence of 3 μm MitMAB to a 35‐mm glass‐bottom dish precoated with Cell‐Tak Cell and Tissue Adhesive. Stock solutions of 3 mm MitMAB were prepared by dissolving in α‐MEM. Erythroblasts were then cultured in the presence or absence of holo‐Tf or hinokitiol plus iron at 37 °C for 30 min. After washing with ice‐cold PBS (−), erythroblasts were fixed in PBS (−) containing 4% paraformaldehyde for 15 min at room temperature and then permeabilized using PBS (−) containing 0.1% Triton X‐100 for 10 min at room temperature. The erythroblasts were then stained with the anti‐TfR1 antibody for 1 h at room temperature. After washing, erythroblasts were stained with RITC‐conjugated anti‐rabbit IgG antibody, mounted in Prolong Glass Antifade Mountant (Thermo Fisher Scientific), and observed using a laser scanning confocal microscope (A1R; Nikon, Tokyo, Japan).

### Statistical analyses

All statistical analyses were performed using Student's *t*‐test or one‐ or two‐way ANOVA, followed by Tukey's test. All data represent the mean ± SEM. Statistical significance is expressed as follows: *P < 0.05; **p < 0.01; ***p < 0.001, or not significant (NS).

## Results

### Flow cytometry analysis of mouse spleen‐derived erythroblast enucleation

**Figure 1:**
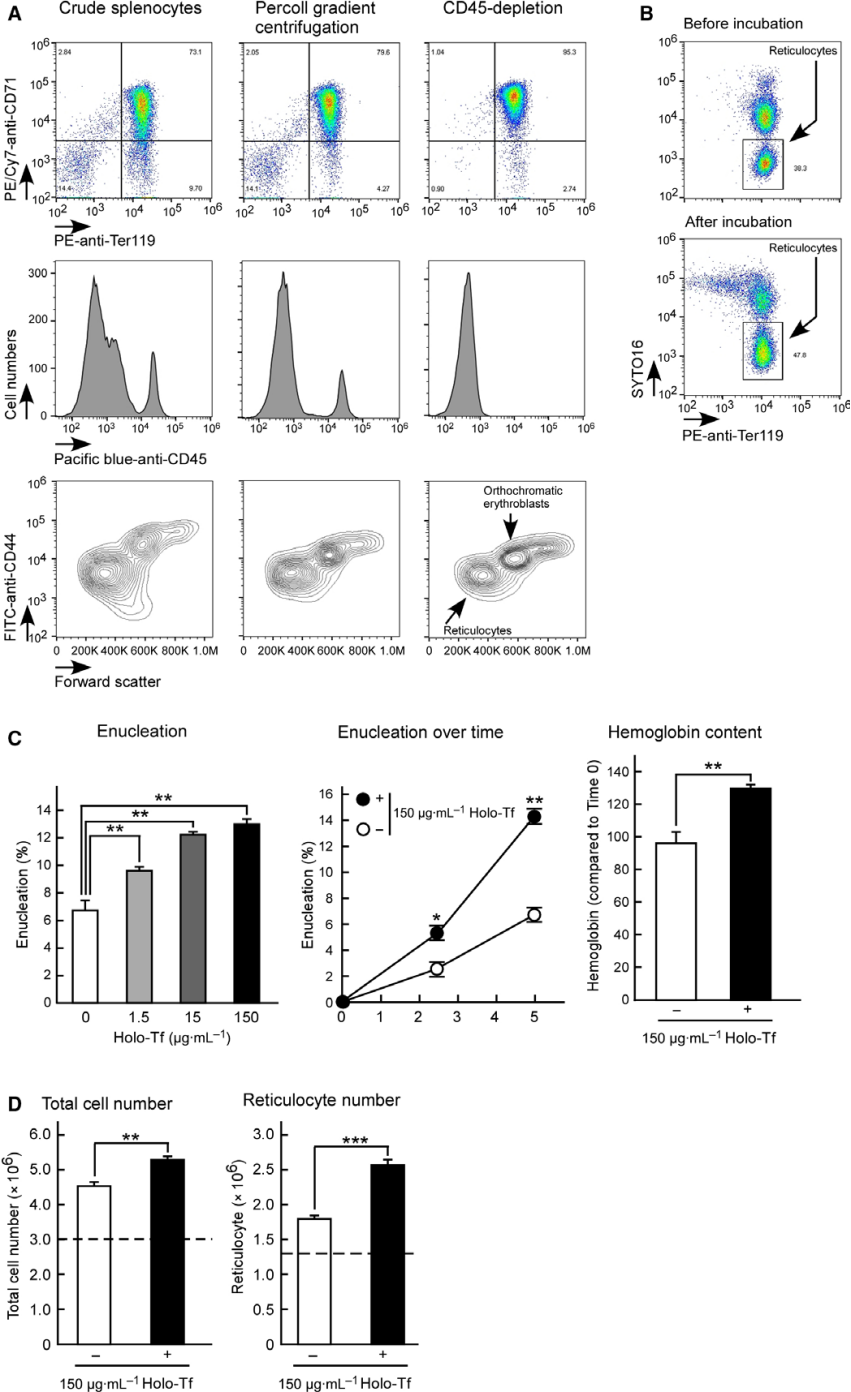
Holo‐Tf stimulates enucleation and hemoglobin synthesis in mouse spleen‐derived erythroblasts via TfR1. (A) Crude splenocytes and splenocytes after Percoll gradient centrifugation and subsequent CD45‐depletion were stained for PE‐anti‐Ter119, PE/Cy7‐anti‐CD71, and Pacific blue‐anti‐CD45 antibodies and analyzed by flow cytometry. Ter119‐positive cells in each fraction were further analyzed using FITC‐anti‐CD44 antibody. Purified CD45‐negative cells were used as mouse spleen‐derived erythroblast preparation. (B) Mouse spleen‐derived erythroblasts before or after incubation for 5 h were stained with PE‐anti‐Ter119 and SYTO16 and then analyzed by flow cytometry. (C) Enucleation and hemoglobin synthesis assays of mouse spleen‐derived erythroblasts in the presence of various concentrations of human holo‐Tf (one‐way ANOVA). Erythroblasts were incubated alone (open circles) or with 150 μg·mL^−1^ of human holo‐Tf (closed circles) for the indicated time (two‐way ANOVA). Purified mouse spleen‐derived erythroblasts were incubated for 5 h. Then, their hemoglobin content was quantified (Student's *t*‐test). (D) The total cell numbers (×10^6^) and the number of reticulocytes after 5 h of culture in the presence/absence of holo‐Tf (Student's *t*‐test). The dashed lines show the starting cell number (3.0 × 10^6^) and the initial number of reticulocytes (1.3 × 10^6^), respectively. All experiments were repeated four times. (C, D) All data are the mean ± SEM. *P < 0.05, **P < 0.01, and ***p < 0.001 (compared to the control).

To study the molecular mechanisms underlying erythroid enucleation, we performed *in vitro* enucleation assays of primary mouse spleen‐derived erythroblasts. A large number of erythroblasts can be collected using this system 17, 19. When the single‐cell suspension was prepared from spleens 6 days after the 48‐h administration of phenylhydrazine, 78.3% ± 2.6% of the single‐cell suspension comprised CD71^high^Ter119high erythroid cells (Fig. 1A). Cells were then separated on a 70% Percoll gradient to reduce the number of CD71^low^Ter119^high^ mature erythrocytes. The resulting cells were CD71^high^Ter119^high^ erythroid cells (81.3% ± 0.9%; Fig. 1A). CD45 is known as a nonerythroid cell marker. To remove the nonerythroid cells from the suspension, CD45‐positive cells were excluded from the cell suspension using magnetic separation 18, 21. The resulting purified CD45‐negative cells were CD71^high^Ter119^high^ erythroid cells with a purity of 95.2% ± 0.1% (Fig. 1A). Staining with the anti‐CD44 antibody revealed that the purified CD45‐negative cells primarily represented orthochromatic erythroblasts and reticulocytes (Fig. 1A). These purified cells were then used as mouse spleen‐derived erythroblasts for studying the enucleation process. To assess enucleated erythroblasts in mouse spleen‐derived erythroblasts, cells were stained with SYTO16, a cell‐permeable DNA‐staining dye, and the PE‐anti‐mouse Ter119 antibody. SYTO16^low^Ter119^high^ cells represented reticulocytes (Fig. 1B).

### Holo‐Tf promotes erythroblast enucleation via TfR1

Transferrin is an iron‐carrying blood plasma glycoprotein responsible for Fe^3+^ delivery from sites of absorption and storage to tissue cells 22, 23. When erythroblasts are cultured, Tf is usually added to the medium to supply iron to erythroblasts, although bovine fetal serum also contains some Tf. In this study, we first cultivated mouse spleen‐derived erythroblasts in serum‐free medium in the presence/absence of human holo‐Tf to investigate the effect of holo‐Tf on erythroblast enucleation. Five hours later, the erythroblasts were collected and the extent of enucleation was evaluated by flow cytometry (Fig. 1B). We found that human holo‐Tf stimulated hemoglobin synthesis and erythroblast enucleation in a concentration‐ and time‐dependent manner (Fig. 1C). Next, we measured the number of total cells or reticulocytes after culture in the presence/absence of holo‐Tf (Fig. 1D). Our results indicated that the absolute number of reticulocytes increased in the presence of holo‐Tf.

**Figure 2:**
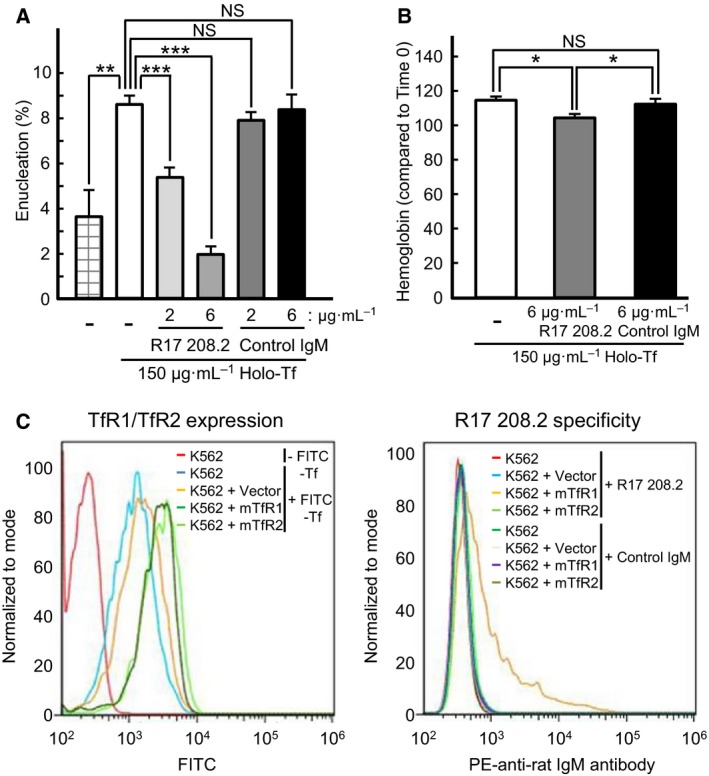
The anti‐TfR1 monoclonal antibody R17 208.2 blocks mouse erythroblast enucleation. (A) Enucleation assays of primary mouse spleen‐derived erythroblasts in the presence of 150 μg·mL^−1^ of human holo‐Tf and various concentrations of the R17 208.2 antibody or control IgM. (B) Hemoglobin assays of mouse spleen‐derived erythroblasts in the presence of 150 μg·mL^−1^ of human holo‐Tf and 6 μg·mL^−1^ of the R17 208.2 antibody or control IgM. (A, B) Experiments were repeated four times (one‐way ANOVA). All data are the mean ± SEM. *p < 0.05, **p < 0.01, and ***p < 0.001 (compared to the control). (C) Confirmation of mouse TfR1 or TfR2 expression on the cell surface of K562 cells by staining with FITC‐labeled human holo‐Tf. Analysis of the specificity of the R17 208.2 antibody. Parental K562, pcDNA3.1‐K562, mTfR1‐K562, and mTfR2‐K562 cells were stained with either the R17 208.2 antibody or control IgM, followed with the PE‐anti‐Rat IgM antibody. Then, cells were analyzed by flow cytometry. The R17 208.2 antibody recognized TfR1 but not TfR2.

The first step in iron transport to erythroblasts by Tf is the binding of Tf to a specific cell surface receptor, such as TfR1 or TfR2. We next examined whether TfR1 mediated the holo‐Tf‐promoted enucleation mechanism using the anti‐TfR1 antibody R17 208.2, which recognizes TfR1. Mouse spleen‐derived erythroblasts were incubated with the R17 208.2 antibody or control antibody (control IgM) at 4 °C for 30 min and then cultured for 5 h in the presence of 150 μg·mL^−1^ of holo‐Tf. The R17 208.2 antibody, but not the control antibody, inhibited hemoglobin synthesis and enucleation in a concentration‐dependent manner (Fig. 2A,B), suggesting that holo‐Tf‐promoted erythroblast enucleation was mediated by TfR1.

Transferrin receptors 1 and 2 are expressed in erythroid precursor cells or erythroblasts 24. It is not known whether the R17 208.2 antibody recognizes mouse TfR2, in addition to mouse TfR1. To test this possibility, we investigated whether the antibody would recognize overexpressed mouse TfR2. To this end, the human erythroleukemia cell line K562 was stably transfected with the pcDNA3.1 vector alone or with pcDNA3.1 carrying cDNA encoding mouse TfR1 or TfR2. To confirm the overexpression of mouse TfR1 and TfR2 on the surface of the transfectants, cells were incubated with 150 μg·mL^−1^ of biotinylated holo‐Tf on ice, followed by incubation with streptavidin–FITC. Tf binding to the surface of the transfectants was then assessed using flow cytometry. Significant levels of Tf bound to parental K562 cells or pcDNA3.1 transfectants (Fig. 2C) were observed because holo‐Tf bound to human TfR originally expressed in the K562 cells. Conversely, higher levels of holo‐Tf binding were identified in K562 cells that expressed mouse TfR1 or mouse TfR2. These results revealed that mouse TfR1 and TfR2 were expressed on the surface of the respective transfectants. All cells were then incubated with the R17 208.2 antibody or control rat IgM on ice for 15 min, followed by incubation with the PE‐labeled anti‐rat IgM antibody. The fluorescence of PE bound to each transfectant was then measured using flow cytometry. Our results verified that the R17 208.2 antibody recognized mouse TfR1, but not mouse TfR2 (Fig. 2C), suggesting that holo‐Tf promoted erythroblast enucleation via TfR1.

### Hinokitiol plus iron stimulates mouse spleen‐derived erythroblast enucleation and hemoglobin synthesis, while the anti‐TfR1 antibody suppresses only enucleation

**Figure 3:**
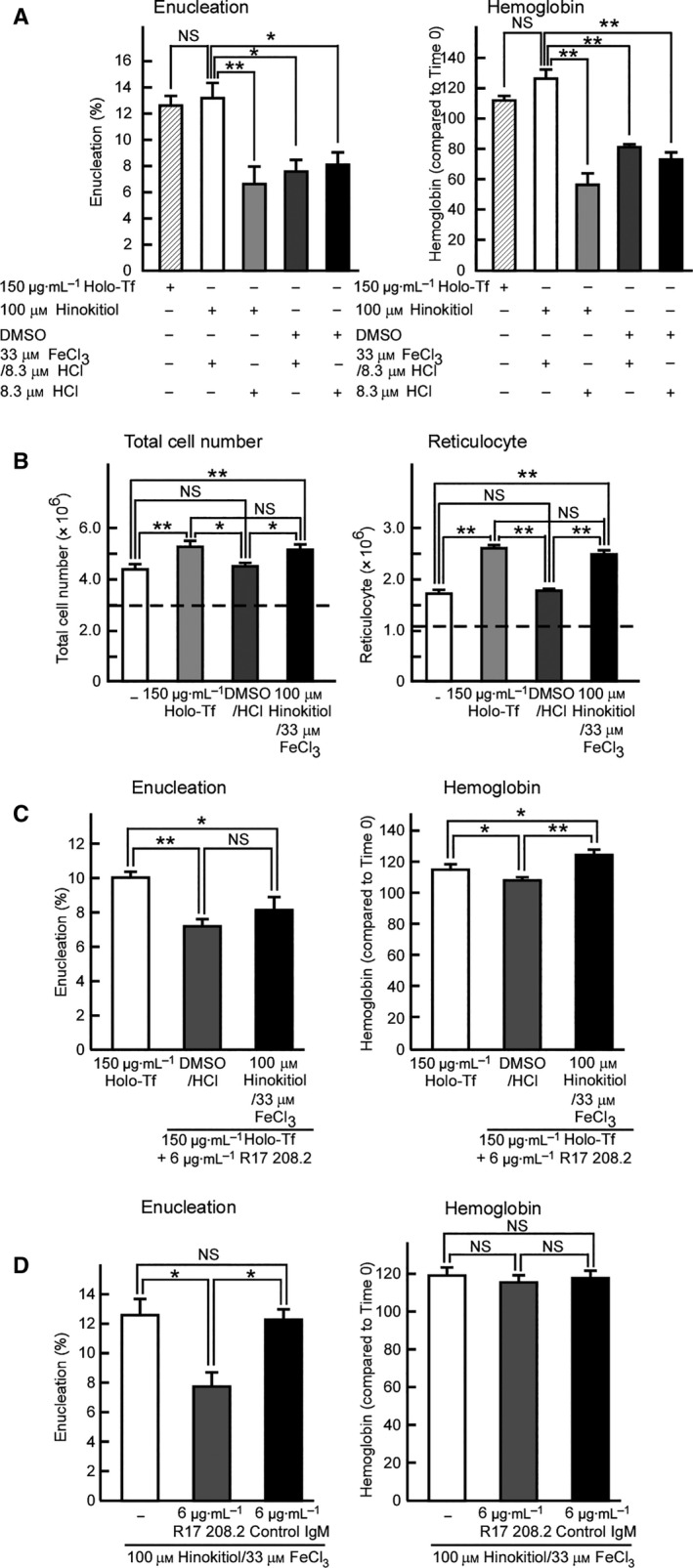
Hinokitiol plus iron stimulates mouse spleen‐derived erythroblast enucleation. (A) Enucleation or hemoglobin assays of mouse spleen‐derived erythroblasts in the presence of 100 μm hinokitiol, 33 μm FeCl_3_, vehicle, or both 100 μm hinokitiol and 33 μm FeCl_3_. Experiments were performed eight or four times (one‐way ANOVA). (B) The total cell numbers (×10^6^) and the number of reticulocytes after 5 h of culture in the presence/absence of hinokitiol plus iron. The dashed lines show the starting cell number (3.0 × 10^6^) and the initial number of reticulocytes (1.1 × 10^6^), respectively. Experiments were performed six times (one‐way ANOVA). (C) Hinokitiol plus iron did not negate the inhibitory effect of the R17 208.2 antibody (6 μg·mL^−1^) on 150 μg·mL^−1^ holo‐Tf‐promoted enucleation, but canceled the inhibitory effect on holo‐Tf‐promoted hemoglobin synthesis. Experiments were performed eight or 13 times (one‐way ANOVA). (D) The R17 208.2 antibody (6 μg·mL^−1^) inhibited 100 μm hinokitiol and 33 μm FeCl_3_‐promoted mouse spleen‐derived erythroblast enucleation, but not hemoglobin synthesis. Experiments were performed six times (one‐way ANOVA). (A–D) All data are the mean ± SEM. *p < 0.05 and **p < 0.01 (compared to the control).

Stimulation of mouse spleen‐derived erythroblast enucleation by holo‐Tf suggested that iron ions play a crucial role in erythroblast enucleation. To test this possibility, we utilized the natural product hinokitiol, which binds to iron ions and transports them into cells through the plasma membrane 16. First, we investigated whether hinokitiol plus iron could induce hemoglobin synthesis and erythroblast enucleation in the absence of holo‐Tf. Our results indicated that hinokitiol plus iron ions induced enucleation and hemoglobin synthesis; however, hinokitiol or iron ions alone did not (Fig. 3A). To confirm the stimulation of erythroblast enucleation by hinokitiol plus iron, we directly measured the number of total cells or reticulocytes after culture in the presence/absence of hinokitiol and iron ions (Fig. 3B); consequently, hinokitiol plus iron increased the number of enucleated erythroblasts. Collectively, our results indicated that iron ions play a crucial role in erythroblast enucleation.

We then examined whether hinokitiol plus iron could cancel the inhibitory effect of the R17 208.2 antibody on holo‐Tf‐promoted enucleation. Hinokitiol plus iron was indeed shown to negate the inhibitory effect on Tf‐promoted hemoglobin synthesis by the R17208.2 antibody, but it did not cancel the inhibitory effect on enucleation (Fig. 3C). These findings suggested that the role of TfR1 in mouse erythroblast enucleation is not limited to supplying iron ions. We found that erythroblast enucleation, but not hemoglobin synthesis, promoted by hinokitiol plus iron was strongly inhibited by the R17 208.2 antibody (Fig. 3D). These results indicate that TfR1 is important in holo‐Tf‐ as well as hinokitiol plus iron‐promoted mouse erythroblast enucleation and that the role of TfR1 in erythroblast enucleation is not limited to the supply of iron ions to erythroblasts.

### The anti‐TfR1 antibody blocks mouse fetal liver‐derived erythroblast enucleation

**Figure 4:**
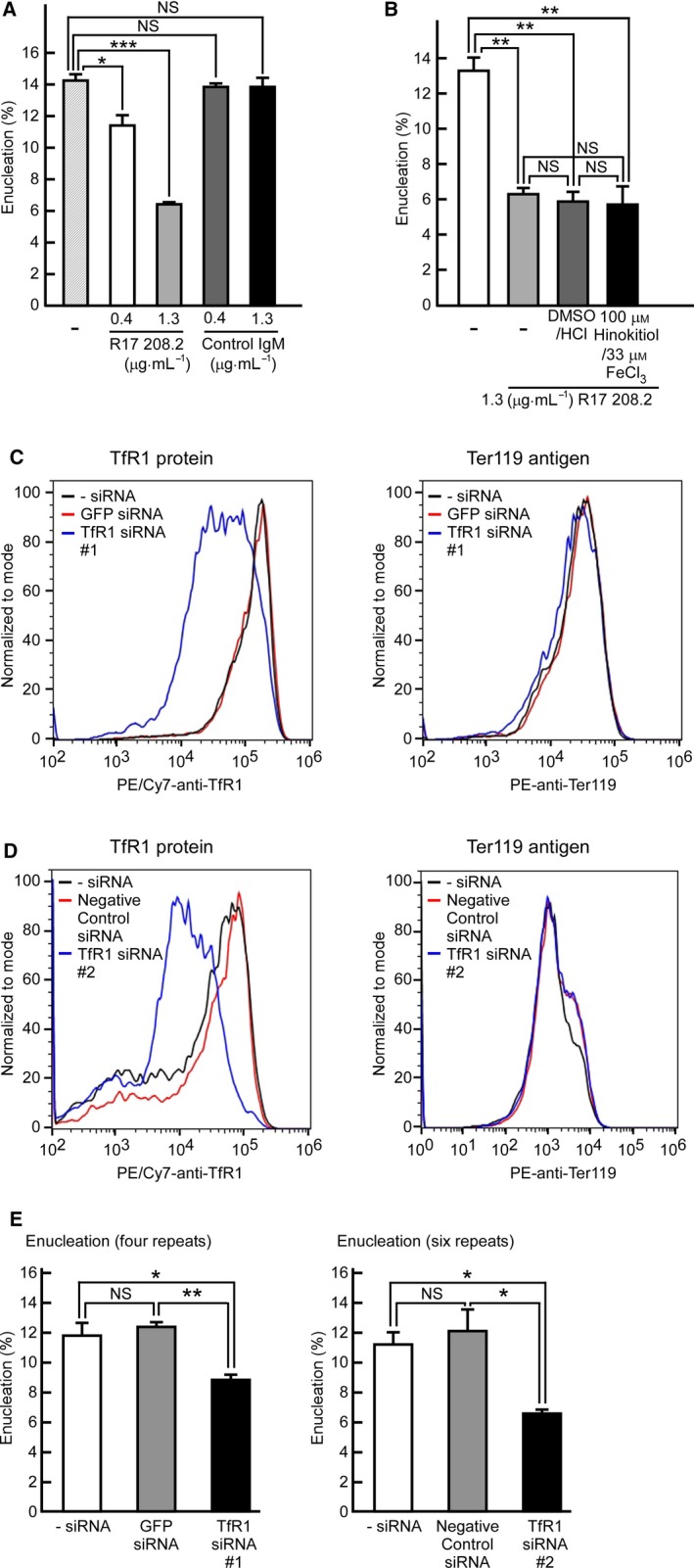
The anti‐TfR1 monoclonal antibody R17 208.2 and siRNA for mouse TfR1 block mouse fetal liver‐derived erythroblast enucleation. (A) Results of enucleation assays of Ter119‐negative mouse fetal liver‐derived erythroblasts in medium containing 20% FBS and various concentrations of the R17 208.2 antibody or control IgM. Experiments were repeated four times (one‐way ANOVA). (B) Hinokitiol plus iron did not cancel the inhibitory effect of the R17 208.2 antibody (1.3 μg·mL^−1^) on mouse fetal liver‐derived erythroblast enucleation. Experiments were performed six times (one‐way ANOVA). (C) Mouse fetal liver‐derived erythroblasts were transfected with mouse TfR1 siRNA #1, #2, the GFP siRNA or negative control siRNA. Expression of mouse TfR1 protein or Ter119 antigen was assessed by flow cytometry with PE/Cy7‐anti‐TfR1 or PE‐anti‐Ter119. (D, E) Mouse fetal liver‐derived erythroblasts were transfected with mouse TfR1 siRNA #1, mouse TfR1 siRNA #2, the GFP siRNA or negative control siRNA, and then were cultured for 48 h. The ratio of enucleation was analyzed by flow cytometry. Experiments were repeated four times or six times (one‐way ANOVA). All data are the mean ± SEM. *p < 0.05, **p < 0.01, and ***p < 0.001 (compared to the control).

To investigate whether TfR1 also plays a major role in mouse fetal liver‐derived erythroblast enucleation, we examined whether the R17 208.2 antibody suppressed mouse fetal liver‐derived erythroblast enucleation. However, we were not able to measure the enucleation efficiency of holo‐Tf or hinokitiol plus iron in serum‐free medium because the primary mouse fetal liver‐derived erythroblasts failed to survive in serum‐free medium. Since the addition of bovine holo‐Tf to the serum‐free medium promoted mouse spleen‐derived erythroblast enucleation, we believed that FBS containing bovine holo‐Tf would promote mouse fetal liver‐derived erythroblast enucleation. Mouse fetal liver‐derived erythroblasts were cultured for 30 h in the presence of EPO; erythroblasts were then cultured with the R17 208.2 antibody or control antibody for an additional 18 h. The R17 208.2 antibody inhibited fetal liver‐derived erythroblast enucleation, whereas the control antibody did not (Fig. 4A). We next investigated whether hinokitiol plus iron could cancel the inhibitory effect of the R17 208.2 antibody on mouse fetal liver‐derived erythroblast enucleation. As shown in Fig. 4B, hinokitiol plus iron did not negate the inhibition effect on enucleation. These results indicate that TfR1 plays an important role in fetal liver‐derived erythroblast enucleation.

### siRNA for mouse TfR1 suppresses the enucleation of mouse fetal liver‐derived erythroblasts

Purified mouse fetal liver‐derived erythroblasts were transfected with mouse TfR1 siRNA, and the expression of mouse TfR1 on the cell surface was examined by flow cytometry after 30‐h culture. The TfR1 protein on the cell surface was reduced in the TfR1 siRNA‐transfected cells compared with the GFP siRNA‐transfected cells or nontransfected control (Fig. 4C). On the other hand, the expression of the Ter119 antigen, a differentiation marker of mature erythroblasts, was not affected by TfR1 siRNA or GFP siRNA (Fig. 4C). After an additional 18 h, inhibition of enucleation was observed only in the TfR1 siRNA‐transfected erythroblasts (Fig. 4D). Similar results were obtained when another TfR1 siRNA were used, confirming a crucial role of TfR1 in the enucleation of erythroblasts (Fig. 4C,D).

### The endocytosis inhibitor MitMAB inhibits enucleation, hemoglobin synthesis, and the internalization of TfR1 promoted by either holo‐Tf or hinokitiol plus iron

**Figure 5:**
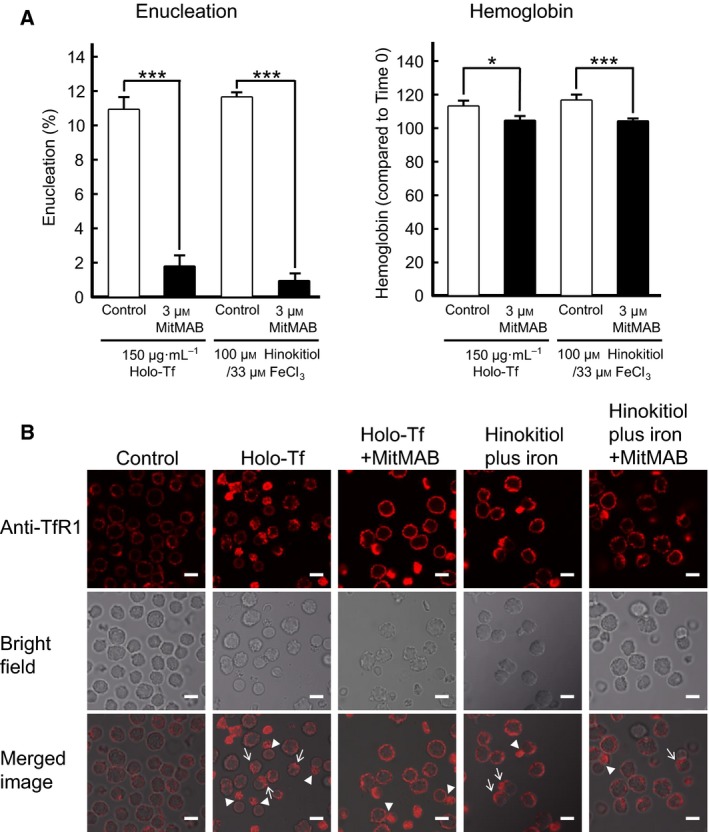
The endocytosis inhibitor MitMAB blocked enucleation, hemoglobin synthesis of mouse spleen‐derived erythroblasts, and the internalization of TfR1. (A) Enucleation or hemoglobin assays of mouse spleen‐derived erythroblasts in the absence or presence of 3 μm MitMAB. Experiments were performed six times (Student's *t*‐test). All data are the mean ± SEM. *p < 0.05, and ***p < 0.001 (compared to the control). (B) The endocytosis of TfR1 induced by holo‐Tf or hinokitiol plus iron was suppressed in the presence of dynamin inhibitor MitMAB. Mouse spleen‐derived erythroblasts were stimulated by holo‐Tf or hinokitiol plus iron in the presence or absence of 3 μm MitMAB, and 30 min later, the erythroblasts were immunostained with the anti‐TfR1 antibody followed by the RITC‐conjugated anti‐rabbit antibody (Top panel). The bright field images and the merged images are shown in middle panels and bottom panels, respectively. Arrows indicate the localization of TfR1 to the opposite side of the nucleus. Arrowheads indicate the localization of TfR1 to the cytoplasmic side immediately after enucleation. MitMAB suppressed the change of localization of TfR1 occurred after both stimulations. Scale bar, 10 μm.

Previous reports have suggested that Tf is located in the cytoplasm adjacent to the extruding nuclei during the enucleation of erythroblasts 12, 25. It may be that TfR1 is transported from the cell surface into the cell by endocytosis during erythroblast enucleation. To investigate whether this indeed occurs, we examined the effect of the endocytosis inhibitor MitMAB on enucleation and hemoglobin synthesis in mouse spleen‐derived erythroblasts promoted by holo‐Tf or hinokitiol plus iron (Fig. 5A). MitMAB inhibited enucleation and hemoglobin synthesis promoted by either holo‐Tf or hinokitiol plus iron. The internalization of TfR1 was further examined using immunofluorescence microscopy after staining the cells for mouse TfR1 (Fig. 5B). TfR1 localized on the plasma membrane before stimulation and accumulated on the cytoplasmic side, not the nucleus side, of enucleating cells after stimulation by holo‐Tf or hinokitiol. These results suggested that endocytosis is important for the enucleation of mouse spleen‐derived erythroblasts and is necessary for the effects of hinokitiol plus iron, as well as those of holo‐Tf.

## Discussion

In this study, we demonstrated that the siRNA for mouse TfR1 and the anti‐mouse TfR1 monoclonal antibody R17 208.2 suppressed mouse erythroblast enucleation (Figs 2, 3, 4). Holo‐Tf binds to the cell surface receptor TfR and supplies iron ions to the cells via holo‐Tf–TfR complex internalization by endocytosis 22. How does Tf promote erythroblast enucleation? Our findings indicate that Tf promotes receptor‐mediated endocytosis. Electron microscopy of enucleating canine erythroblasts has revealed that many vesicles accumulate in the cytoplasm adjacent to the envelope of the extruding nucleus 26. It was also observed that a series of coalesced vesicles combined with another series of coalesced vesicles to form a larger single vesicle. Eventually, a series of coalesced vesicles completely undermined the circumference of the nucleus not associated with the cell membrane, at which point the nucleus was completely pinched off from the erythroblast. Iacopetta *et al*. 25 also reported that Tf is taken up in cells by receptor‐mediated endocytosis and accumulates in the cytoplasm adjacent to the extruding nucleus in mouse erythroblasts. Keerthivasan *et al*. 12 demonstrated that the inhibition of vesicle trafficking or knockdown of clathrin, a protein that plays a major role in the formation of coated vesicles, blocks mouse or human erythroblast enucleation. In addition, the authors showed that treatment with vacuolin‐1, which promotes the formation of enlarged vacuoles, increases mouse erythroblast enucleation. Survivin, an inhibitor of the apoptosis family of proteins, is abundantly expressed in orthochromatic erythroblasts 27. Keerthivasan *et al*. 13 demonstrated that survivin binds to clathrin and EPS15, which are two proteins that mediate endocytic vesicle trafficking. The authors further evidenced that the knockdown of survivin or EPS15 significantly inhibits enucleation without affecting the survival or differentiation of human erythroblasts. Furthermore, the R17 208.2 antibody has been reported not to block Tf from binding to TfR1, but rather blocks TfR1 internalization 28, 29. In this study, the endocytosis inhibitor MitMAB inhibited mouse erythroblast enucleation promoted by holo‐Tf (Fig. 5A). Furthermore, MitMAB suppressed TfR1 internalization after stimulation with holo‐Tf (Fig. 5B). Our results, together with those of previous reports, suggest that holo‐Tf promotes erythroblast enucleation via the enhancement of receptor‐mediated endocytosis, in addition to the supply of iron ions.

Transferrin receptor 2 is another member of TfRs with a moderate homology to TfR1. TfR2 is capable of binding to holo‐Tf, although its affinity for holo‐Tf is lower 30. We found that the R17 208.2 antibody specifically binds to TfR1, but not to TfR2 (Fig. 2C). A major role of TfR1 in holo‐Tf‐promoted enucleation was confirmed by our study using siRNA for mouse TfR1, which suppressed the enucleation of mouse fetal liver‐derived erythroblasts (Fig. 4D). These results suggest that TfR2 does not play a major role in holo‐Tf‐promoted enucleation.

Hinokitiol can transport iron ions into cells through the plasma membrane. Similar to holo‐Tf, when hinokitiol plus iron was added to mouse spleen‐derived erythroblasts, enucleation was promoted, while this effect was inhibited by the R17 208.2 antibody (Fig. 3D). Moreover, the endocytosis inhibitor MitMAB inhibited erythroblast enucleation and the intracellular accumulation of the TfR1 protein (Fig. 5A,B). These results suggested that TfR1‐dependent endocytosis might play a role in the enucleation promoted by hinokitiol plus iron. Since the R17208.2 antibody did not suppress hemoglobin synthesis promoted by hinokitiol plus iron (Fig. 3D), it was suggested that hemoglobin synthesis stimulated by hinokitiol plus iron depends on TfR1‐independent endocytosis. Esparza *et al*. 31 reported that iron‐induced reactive oxygen species mediate endocytosis in intestinal epithelial cells. Therefore, we propose that iron ions were incorporated into erythroblasts in the form of hinokitiol plus iron‐induced reactive oxygen species and then the reactive oxygen species induced endocytosis. In both cases, the R17 208.7 antibody may have inhibited endocytosis by immobilizing TfR1 on the plasma membrane and inhibiting enucleation.

Enucleation is an event specific to mammalian erythroblasts; Tf and TfR1 exist in other vertebrates, as well as in mammals, but only mammalian erythroblasts release their nuclei. Although human TfR shows high expression in human erythroleukemia K562 cells, when we added holo‐Tf to the cells, the holo‐Tf bound to the surface of the K562 cells but enucleation did not occur (data not shown). This may be due to specific intracellular signal transduction pathways involved from the uptake of Tf to enucleation that are present in mammalian erythroblasts, but not in K562 cells. To understand the molecular mechanisms underlying enucleation in mammalian erythroblasts, further studies of the role of vesicle transport by endocytosis in enucleation are warranted.

## Conflicts of interest

The authors declare no conflicts of interest.

## Author contributions

MA designed and performed the experiments, interpreted data, and drafted the manuscript. AI, KM, and NO provided assistance with the experiments and analyzed data. YT and NM assisted with the experimental design and edited the manuscript.

## References

[1] Wu H , Liu X , Jaenisch R and Lodish HF (1995) Generation of committed erythroid BFU‐E and CFU‐E progenitors does not require erythropoietin or the erythropoietin receptor. Cell 83, 59–67. 7553874 10.1016/0092-8674(95)90234-1

[2] Koury MJ and Bondurant MC (1988) Maintenance by erythropoietin of viability and maturation of murine erythroid precursor cells. J Cell Physiol 137, 65–74. 2459142 10.1002/jcp.1041370108

[3] Ji P , Yeh V , Ramirez T , Murata‐Hori M and Lodish HF (2010) Histone deacetylase 2 is required for chromatin condensation and subsequent enucleation of cultured mouse fetal erythroblasts. Haematologica 95, 2013–2021. 20823130 10.3324/haematol.2010.029827 PMC2995558

[4] Li X , Mei Y , Yan B , Vitriol E , Huang S , Ji P and Qiu Y (2017) Histone deacetylase 6 regulates cytokinesis and erythrocyte enucleation through deacetylation of formin protein mDia2. Haematologica 102, 984–994. 28255013 10.3324/haematol.2016.161513 PMC5451330

[5] Ji P , Jayapal SR and Lodish HF (2008) Enucleation of cultured mouse fetal erythroblasts requires Rac GTPases and mDia2. Nat Cell Biol 10, 314–321. 18264091 10.1038/ncb1693

[6] Ubukawa K , Guo YM , Takahashi M , Hirokawa M , Michishita Y , Nara M , Tagawa H , Takahashi N , Komatsuda A , Nunomura W *et al* (2012) Enucleation of human erythroblasts involves non‐muscle myosin IIB. Blood 119, 1036–1044. 22049517 10.1182/blood-2011-06-361907 PMC3352306

[7] Konstantinidis DG , Pushkaran S , Johnson JF , Cancelas JA , Manganaris S , Harris CE , Williams DA , Zheng Y and Kalfa TA (2012) Signaling and cytoskeletal requirements in erythroblast enucleation. Blood 119, 6118–6127. 22461493 10.1182/blood-2011-09-379263 PMC3383020

[8] Eshghi S , Vogelezang MG , Hynes RO , Griffith LG and Lodish HF (2007) Alpha4beta1 integrin and erythropoietin mediate temporally distinct steps in erythropoiesis: integrins in red cell development. J Cell Biol 177, 871–880. 17548514 10.1083/jcb.200702080 PMC2064286

[9] Wang J , Ramirez T , Ji P , Jayapal SR , Lodish HF and Murata‐Hori M (2012) Mammalian erythroblast enucleation requires PI3K‐dependent cell polarization. J Cell Sci 125, 340–349. 22331356 10.1242/jcs.088286 PMC3283871

[10] Zhang L , Flygare J , Wong P , Lim B and Lodish HF (2011) miR‐191 regulates mouse erythroblast enucleation by down‐regulating Riok3 and Mxi1. Genes Dev 25, 119–124. 21196494 10.1101/gad.1998711 PMC3022257

[11] Wang C , Wu X , Shen F , Li Y , Zhang Y and Yu D (2015) Shlnc‐EC6 regulates murine erythroid enucleation by Rac1‐PIP5K pathway. Dev Growth Differ 57, 466–473. 26098172 10.1111/dgd.12225

[12] Keerthivasan G , Small S , Liu H , Wickrema A and Crispino JD (2010) Vesicle trafficking plays a novel role in erythroblast enucleation. Blood 116, 3331–3340. 20644112 10.1182/blood-2010-03-277426 PMC2995360

[13] Keerthivasan G , Liu H , Gump JM , Dowdy SF , Wickrema A and Crispino JD (2012) A novel role for survivin in erythroblast enucleation. Haematologica 97, 1471–1479. 22491741 10.3324/haematol.2011.061093 PMC3487547

[14] Anderson CP , Shen M , Eisenstein RS and Leibold EA (2012) Mammalian iron metabolism and its control by iron regulatory proteins. Biochim Biophys Acta 1823, 1468–1483. 22610083 10.1016/j.bbamcr.2012.05.010 PMC3675657

[15] Dautry‐Varsat A , Ciechanover A and Lodish HF (1983) pH and the recycling of transferrin during receptor‐mediated endocytosis. Proc Natl Acad Sci USA 80, 2258–2262. 6300903 10.1073/pnas.80.8.2258 PMC393798

[16] Grillo AS , SantaMaria AM , Kafina MD , Cioffi AG , Huston NC , Han M , Seo YA , Yien YY , Nardone C , Menon A V *et al* (2017) Restored iron transport by a small molecule promotes absorption and hemoglobinization in animals. Science (80‐.) 356, 608–616. 10.1126/science.aah3862 PMC5470741 28495746

[17] Yoshida H , Kawane K , Koike M , Mori Y , Uchiyama Y and Nagata S (2005) Phosphatidylserine‐dependent engulfment by macrophages of nuclei from erythroid precursor cells. Nature 437, 754–758. 16193055 10.1038/nature03964

[18] Liu J , Zhang J , Ginzburg Y , Li H , Xue F , De FL , Chasis JA , Mohandas N and An X (2015) Quantitative analysis of murine terminal erythroid differentiation *in vivo*: novel method to study normal and disordered erythropoiesis. Blood 121, 43–50. 10.1182/blood-2012-09-456079 PMC3578961 23287863

[19] Chow A , Huggins M , Ahmed J , Hashimoto D , Lucas D , Kunisaki Y , Pinho S , Leboeuf M , Noizat C , van Rooijen N *et al* (2013) CD169(+) macrophages provide a niche promoting erythropoiesis under homeostasis and stress. Nat Med 19, 429–436. 23502962 10.1038/nm.3057 PMC3983996

[20] Zhang J , Socolovsky M , Gross AW and Lodish HF (2003) Role of Ras signaling in erythroid differentiation of mouse fetal liver cells: functional analysis by a flow cytometry‐based novel culture system. Blood 102, 3938–3946. 12907435 10.1182/blood-2003-05-1479

[21] Han Y , Liu Q , Hou J , Gu Y , Zhang Y , Chen Z , Fan J , Zhou W , Qiu S , Zhang Y *et al* (2018) Tumor‐induced generation of splenic erythroblast‐like Ter‐cells promotes tumor progression. Cell 173, 634–648.e12. 29606356 10.1016/j.cell.2018.02.061

[22] Aisen P and Listowsky I (1980) Iron transport and storage proteins. Annu Rev Biochem 49, 357–393. 6996567 10.1146/annurev.bi.49.070180.002041

[23] Lambert LA (2012) Molecular evolution of the transferrin family and associated receptors. Biochim Biophys Acta 1820, 244–255. 21693173 10.1016/j.bbagen.2011.06.002

[24] Kawabata H , Germain RS , Ikezoe T , Tong X , Green EM , Gombart AF , Koeffler HP , Kawabata H , Germain RS , Ikezoe T *et al* (2011) Regulation of expression of murine transferrin receptor 2. Blood 98, 1949–1954. 10.1182/blood.v98.6.1949 11535534

[25] Iacopetta BJ , Morgan EH and Yeoh GC (1983) Receptor‐mediated endocytosis of transferrin by developing erythroid cells from the fetal rat liver. J Histochem Cytochem 31, 336–344. 6300220 10.1177/31.2.6300220

[26] Simpson CF and Kling JM (1967) The mechanism of denucleation in circulating erythroblasts. J Cell Biol 35, 237–245. 6061718 10.1083/jcb.35.1.237 PMC2107122

[27] Gurbuxani S , Xu Y , Keerthivasan G , Wickrema A and Crispino JD (2005) Differential requirements for survivin in hematopoietic cell development. Proc Natl Acad Sci USA 102, 11480–11485. 16055565 10.1073/pnas.0500303102 PMC1183538

[28] Lesley JF and Schulte RJ (1985) Inhibition of cell growth by monoclonal anti‐transferrin receptor antibodies. Mol Cell Biol 5, 1814–1821. 3018527 10.1128/mcb.5.8.1814-1821.1985 PMC366896

[29] Trowbridge IS , Lesley J and Schulte R (1982) Murine cell surface transferrin receptor: studies with an anti‐receptor monoclonal antibody. J Cell Physiol 112, 403–410. 6290505 10.1002/jcp.1041120314

[30] Kawabata H , Germain RS , Vuong PT , Nakamaki T , Said JW and Koeffler HP (2000) Transferrin receptor 2‐alpha supports cell growth both in iron‐chelated cultured cells and *in vivo* . J Biol Chem 275, 16618–16625. 10748106 10.1074/jbc.M908846199

[31] Esparza A , Gerdtzen ZP , Olivera‐Nappa A , Salgado JC and Núñez MT (2015) Iron‐induced reactive oxygen species mediate transporter DMT1 endocytosis and iron uptake in intestinal epithelial cells. Am J Physiol Cell Physiol 309, C558–C567. 26289753 10.1152/ajpcell.00412.2014

